# Titanium Oxide Formation in TiCoCrFeMn High-Entropy Alloys

**DOI:** 10.3390/ma18020412

**Published:** 2025-01-17

**Authors:** Dominika Przygucka, Adelajda Polkowska, Wojciech Polkowski, Krzysztof Karczewski, Stanisław Jóźwiak

**Affiliations:** 1 Faculty of Advanced Technologies and Chemistry, Military University of Technology, Sylwestra Kaliskiego 2, 00-908 Warsaw, Poland; krzysztof.karczewski@wat.edu.pl (K.K.); stanislaw.jozwiak@wat.edu.pl (S.J.); 2 Łukasiewicz Research Network—Krakow Institute of Technology, Zakopiańska 73 Str., 30-418 Krakow, Poland; adelajda.polkowska@kit.lukasiewicz.gov.pl (A.P.); wojciech.polkowski@kit.lukasiewicz.gov.pl (W.P.)

**Keywords:** high-entropy alloys, solid solution, Laves phase, titanium oxides, rutil, anatase, Magnelli phases

## Abstract

High-entropy materials, characterized by complex chemical compositions, are difficult to identify and describe structurally. These problems are encountered at the composition design stage when choosing an effective method for predicting the final phase structure of the alloy, which affects its functional properties. In this work, the effects of introducing oxide precipitates into the matrix of a high-entropy TiCoCrFeMn alloy to strengthen ceramic particles were studied. The particles were introduced by the ex situ method, such as TiO_2_ in the form of anatase, and by the in situ method, consisting of the reconstruction of CuO into TiO_2_. In both cases, it was assumed that after the homogenization process, carried out at 1000 °C, ceramic precipitates in the rutile phase, commonly considered a stable allotropic form of TiO_2_, would be obtained. However, the microscopic observations and XRD analyses, supported by EDS chemical composition microanalysis and EBSD backscattered electron diffraction, clearly revealed that, regardless of the method of introducing oxides, the final strengthening phase obtained was a mixture of TiO_2_ in the form of anatase with the Magnelli phase of Ti_2_O_3_. In this work, phase reconstruction in the Ti-O system was analyzed using changes in the Gibbs free energy of the identified oxide phases.

## 1. Introduction

The strength of structural materials can be improved using many strengthening methods. These methods include strengthening with particles of the second phase, which are particularly easy to apply when fabricating materials by powder metallurgy. One of the approaches to strengthening a structural material using this method is the introduction of oxides into the alloy matrix, which results in strengthening with dispersion oxides; the materials created in this way are classified as oxide dispersion-strengthened (ODS). The structure of these materials consists of oxides dispersed in a metal matrix [1]. The content of oxides in the material matrix affects the greater content of interphase surfaces between the hard phases of oxide ceramics and the metal matrix, resulting in the strengthening of the material due to the Orowan–Ashby or Mott–Nabaro mechanism, and less often due to shearing of the particle by moving the dislocation [2,3]. In the case of noncoherent oxides, there is no continuity in the lattice planes between the ceramic particles and the matrix, resulting in both planes ending at the phase boundary. This phenomenon results in high interfacial energy, which contributes to hindering dislocation movement. Therefore, the use of these stable dispersion particles of oxide ceramics imparts, among other properties, brittleness and resistance to progressive changes in properties at elevated temperatures, including the inhibition of grain growth [4], ensuring high strength due to hindered slip and dislocation climb [1,5]. Moreover, owing to the stability of oxides in ODS alloys, better creep resistance [6], oxidation, and corrosion resistance are achieved than with conventional alloys [5,7]. Due to the uncontrolled removal of oxide particles by growth, coagulation, and precipitation on the casting surface in the form of an oxide scale, ODS alloys are usually fabricated by powder metallurgy [8].

Alloys strengthened with ODS oxides can be based, for example, on heat-resistant oxides (i.e., Y_2_O_3_, Al_2_O_3_, HfO_2_, ThO_2_). One of the proven ODS materials based on Ni-Cr is the MA754 alloy strengthened with yttria, which was introduced for high-temperature applications such as gas turbine blades [9]. Another example is dispersion-strengthened steels used in fourth-generation nuclear power plants. ODS ferritic steels show significant radiation resistance compared with classical ferritic steels [10]. The most crucial goal in this case is to increase the temperature limit (and thus ensure increased creep resistance) while maintaining the advantages of ferritic-martensitic steel by introducing Y_2_O_3_ and TiO_2_ particles into the microstructure [11]. The use of these materials for reactor cores is being considered, especially for applications in fuel cladding and shielding, because they are among the few materials that can be used under high radiation doses, mainly because of their excellent resistance to swelling [12]. The use of specific materials for the construction of generation IV reactors also requires the possibility of operating at a temperature of 1073 K, in contrast to high-chromium ferritic-martensitic steels, for which the maximum operating temperature range is only 823–873 K [11,13,14].

With the continuous development of materials science, research involving the use of oxide dispersion additives for high-entropy alloys is currently underway. One of the most popular high-entropy alloys, Cantor’s CoCr–FeMnNi alloy [15], was compared in terms of tensile strength and yield strength with the same ODS alloy at room temperature and 1073 K; an increase in these parameters of 30% at room temperature and approximately 70% at 1073 K was demonstrated. Notably, a 70% increase in the analyzed parameters occurred at 800 °C, corresponding to the operating temperature of the fourth-generation nuclear reactor [10,15]. It was also shown that in the Al_0.3_CoCrFeMnNi alloy, the yield strength during compression increased from 0.97 to approximately 1.76 GPa with increasing Y_2_O_3_ content from 0 to 3% [16]. The analyses conducted in [10] revealed that high-entropy alloys dispersion-strengthened with ODS oxides exhibit the highest yield strength compared with the entire spectrum of high-entropy compositions.

The results are promising, and the use of related medium-entropy alloys strengthened with oxide ceramic particles by scientists from the NASA Glenn Research Center has led to the proposal of ODS applications. In this case, the NiCoCr alloy was strengthened with yttrium oxide, resulting in increased creep resistance compared with that of the same material without oxides. A material with these characteristics might be used in environments with extreme operating conditions, including gas turbine components, drive systems, or nuclear energy applications [17].

To develop a group of materials with high potential, high-entropy materials containing oxides in the matrix were designed and tested in this work. Their microstructure should be thoroughly examined to evaluate whether the materials could be used in the applications described above. In this work, attention was given to the quantitative content of oxides, and attempts were made to identify the allotropic forms of oxides occurring after the materials had been subjected to long-term annealing processes.

This can be problematic because oxides undergo reconstruction due to changes in the oxidation state of the primary metal, and so-called Magnelli phases can be formed. An important issue is the identification of the allotropic form of oxides in the structure. The most common form of titanium oxide is the stable rutile phase. However, according to [18,19], rutile is practically the only oxidation product up to a temperature of 923 K, and with increasing temperature, other titanium oxides are formed, including the dominant Ti_2_O_3_ with a corundum structure. However, above 1073 K, TiO_2_ and Ti_2_O_3_, TiO, and Magnelli oxides are also observed. In [18], during high-temperature oxidation of a Ti_6_Al_4_V alloy above 973 K, the occurrence of TiO, Ti_2_O_3_, and Ti_3_O_5_ oxides among corrosion products was confirmed. A comprehensive analysis of the above information in the literature [20,21] assumes that anatase and rutile phases are rebuilt through Magnelli phases and Ti_3_O_5_, which is superconductive at temperatures above 3 K, into titanium corundum Ti_2_O_3_.

## 2. Materials and Methods

### 2.1. Analysis of Oxide Properties and Their Selection as Strengthening Additives for the HEA Matrix

To consider the possibility of strengthening high-entropy alloys by introducing oxide ceramics into the matrix, to improve the mechanical properties of the material, tests were performed to strengthen high-entropy TiCoCrFeMn alloys, as discussed previously [22,23]. In addition to the aforementioned base alloy, two other alloys were designed and fabricated using the powder metallurgy method, adding vol. 5% CuO and vol. 5% TiO_2_, respectively. The choice of oxides to be introduced by the in situ (CuO) and ex situ (TiO_2_) methods was determined by the Ellingham-Richardson diagram for metals reacting with oxygen during oxide formation. This allows for the determination of the oxide stability of individual metals and illustrates the changes in the standard Gibbs free energy as a function of temperature for metals reacting with oxygen during oxide formation [24,25]. From the upper part of this diagram, the reactions of the most “noble” metals occur, including those of Au and Pt, whose oxides are unstable and easily reduced. Moving down the diagram with decreasing Gibbs energy, increasingly reactive metals are shown, whose oxides are more difficult to reduce [26]. To use this diagram in selecting the chemical composition of HEAs to strengthen these alloys with ODS oxides, the possibility of oxide decomposition should be considered at each stage of the production process. It is also necessary to predict whether and which of the constituent elements will form oxides or which element’s oxide should be added to the initial mixture of alloy powders so that, owing to its stability, it does not undergo a decomposition reaction, or, in contrast, it will form more stable oxides of other elements. In addition, the characteristic temperatures for a given oxide used as a material-strengthening phase were considered, constituting the framework for the processes to produce the designed alloys and their operating temperature ranges [27].

**Figure 1:**
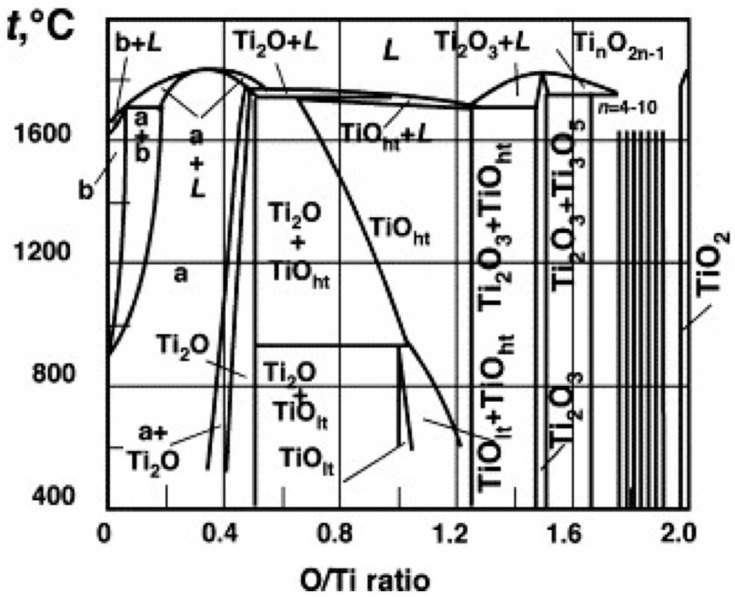
Ti-O equilibrium system made on the basis [32].

Therefore, TiO_2_ oxide was introduced into the structure by the ex situ mechanism because it is not subject to reduction due to its stability; CuO was introduced by the in situ mechanism, and it undergoes reconstruction into stable TiO_2_ when introduced into the structure during the material production stages. The stability of CuO, in contrast to that of TiO_2_, is low because, under the influence of temperature, a two-stage decomposition of this oxide occurs. First, CuO decomposes to Cu_2_O and O_2_ at a higher temperature than Cu_2_O decomposes to Cu and O_2_ [28]. The above properties of CuO allow us to assume that the oxygen released during mechanical alloying, sintering, and annealing reacts with another element with a high affinity for oxygen—in this case, titanium—to create stable titanium oxides during the process. It is assumed that, as a result of the reactions of the oxide added in situ, stable TiO_2_ is formed in the allotropic form of rutile, which is one of the most stable forms of titanium oxides as evidenced by previous works [29,30,31]. The remaining titanium oxides falling into the Magnelli phases are not considered stable; however, owing to the degree of titanium oxidation, many of them can be seen in the equilibrium system (Figure 1).

### 2.2. Production of High-Entropy ODS Alloys

The designed alloys were produced using mechanical alloying and sintering methods. The alloy mixtures were prepared from powders of Co, Cr, Fe, Mn, and Ti metals and TiO_2_ and CuO oxides with a particle size of 45 µm manufactured by Stanford Advanced Materials. The established mixtures of elemental powders were subjected to a 5 h mechanical alloying process in an inert gas atmosphere (argon), which was carried out in a Fritsch Pulverisette 7 Premium planetary ball mill (Fritsch International, Idar-Oberstein, Germany). The ball-to-powder ratio was ~8:1 for one cylinder with a capacity of 80 mL (40 balls of 100 Cr6 bearing steel with a diameter of 10 mm). Then, they were placed in a planetary ball mill and subjected to grinding at a programmed rotational speed of 650 rpm.

After the alloy mixtures were produced, they were sintered and consolidated using the innovative U-FAST volume sintering method. The process was carried out using short current pulses (less than 1 ms) with a high intensity of ~2000 A and a pressure of 13 kN. The mentioned method of sintering metal powders allows the powders to be pressed and simultaneously sintered using short current pulses (less than 1 ms), resulting in a reduction or complete elimination of grain growth [33]. The process was carried out at a heating rate of 50 °C/min at a temperature of 1323 K for one minute; owing to multipoint temperature measurements, it was possible to continuously analyze the shrinkage curve during this process. Then, to homogenize the structure and investigate the effect of the degree of testing the material properties as a function of homogenization time, the samples were annealed in an argon protective atmosphere for 1, 10, 50, 100, and 1000 h at 1273 K. The obtained metallographic samples of alloys in individual states after annealing were subjected to metallographic preparation, which is extremely important in the case of samples subjected to EBSD diffraction tests because, in addition to ensuring a smooth surface free of scratches, this process must lead to the disclosure of grains. The included samples were ground using SiC abrasive papers with gradations from 600 to 4000 and then polished using cloths and a 3 µm and 0.25 µm diamond suspension and a 0.1 µm silica suspension.

X-ray phase analysis (XRD) was performed on a Rigaku Ultima IV X-ray diffractometer (Rigaku, Tokyo, Japan) with a Co radiation source (λ = 1.79 Å) and on a Seifert XRD 3003 TT diffractometer (Seifert, Mannheim, Germany) with a Cu radiation source (λ = 1.54 Å) to identify the phases present in the structure. Microscopic observations of the surface-prepared sample structures were performed using a Quanta 3D FEG scanning electron microscope (SEM) (Field Electron and Ion Company, FEI, Hillsboro, OR, USA), which has a relatively wide range of capabilities. However, using the potential of SE and BSE detectors, the chemical composition of the phases was also analyzed by performing point and line microanalyses of energy dispersive X-ray spectroscopy (EDS). At a later stage, electron backscatter diffraction (EBSD) was used to confirm the crystallographic lattices identified via X-ray diffraction (XRD) and, in the present work, to determine the type of oxides present.

## 3. Results

**Figure 2:**
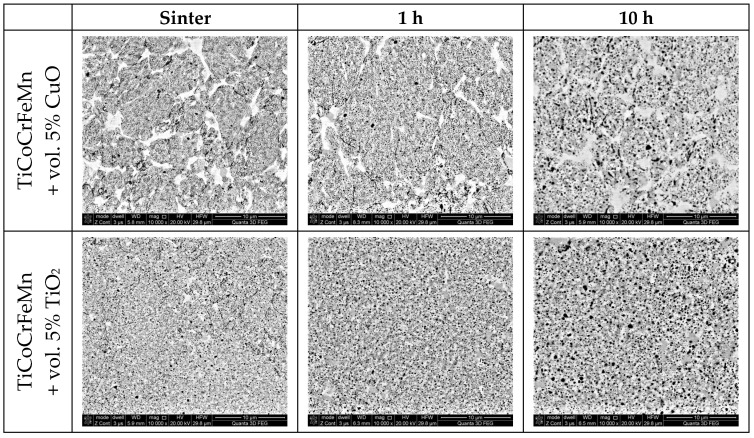

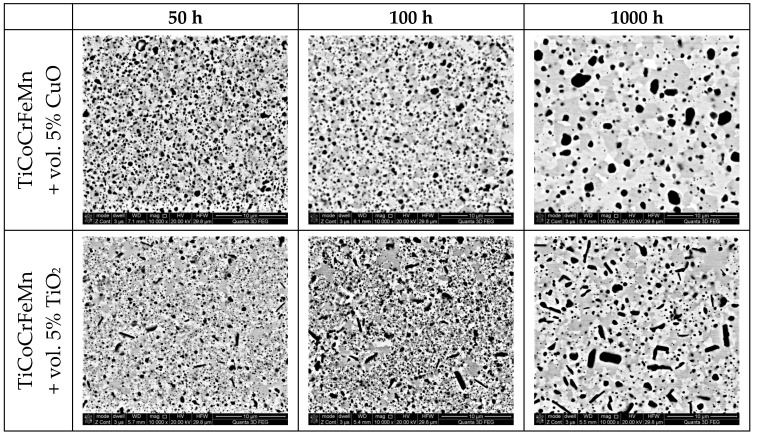
View of the structures of the designed alloys based on TiCoCrFeMn and two alloys based on the addition of 5 vol.% CuO and TiO_2_ after 1000 h of heating at 1273 K.

**Figure 3:**
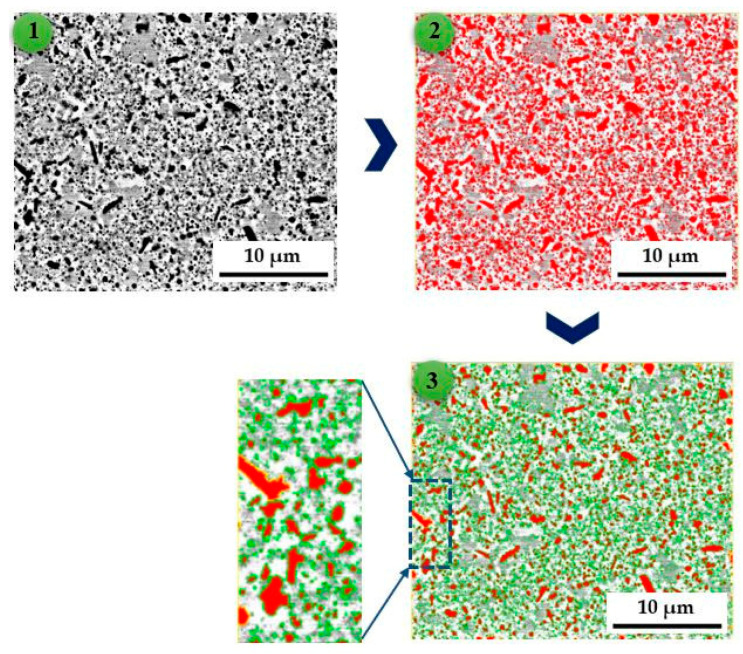
Example of the analysis cycle of the oxide content in the structure of the TiCoCrFeMn alloy + vol. 5% TiO_2_ heated for 100 h (original view of the structure—(**1**); binarized image—(**2**); outlining the of oxides (black precipitations on the original view)—(**3**)).

Microscopic observations of the structure of the sinters, both after the sintering process and after different annealing times at 1273 K in an argon protective atmosphere, clearly revealed changes in the size and morphology of the oxide precipitates with increasing annealing time, regardless of the type of oxide introduced ex situ (Figure 2). With increasing annealing time, the oxides coagulated and grew. Therefore, a more detailed assessment of the content of oxides in the two-phase structure that simultaneously demonstrates the difference between the tested materials is needed. The content of oxides was assessed using NIS Elements BR 5.2 software for computer image analysis. For each material condition, five SEM micrographs were obtained and used from different areas of the sample, where the minimum number of oxides analyzed was over 5000 for samples heated for a long time (fewer oxides of larger sizes) and approximately 20,000 for samples after sintering and short heating times (more oxides of smaller sizes). The counted objects were points for statistical analysis, leading to conclusions on the evolution of oxides across the whole range of homogenization annealing. An example of the image analysis is shown in Figure 3.

**Figure 4:**
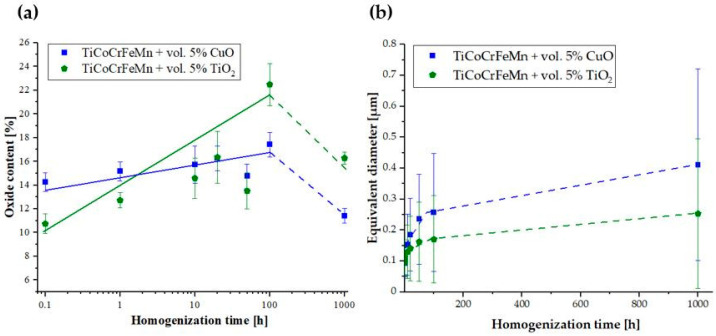
Summary of the oxide fractions (**a**) and their corresponding equivalent diameters (**b**) for the two tested alloys as a function of homogenization time.

The obtained results of the oxide content and equivalent diameter are presented comprehensively as a function of homogenization time along with the standard deviation (Figure 4).

Figure 4 shows that the oxide content of the CuO and TiO_2_ alloys increased after 100 h of homogenization and then decreased after 1000 h of annealing. However, the oxides introduced into the structure by the in situ method, through the reduction of CuO oxide in the presence of TiO_2_, undergo reconstruction to TiO_2_ and grow more slowly, despite being significantly larger, with almost twice the equivalent diameter. The reason may be the greater coherence of the oxide phase with the matrix components during the redox reaction, leading to the reconstruction of oxides. Moreover, the increase in the equivalent diameter of oxides with increasing annealing time is smaller for the TiCoCrFeMn + 5 vol.% TiO_2_ alloy than for the alloy with the addition of CuO. This increase in both alloys takes place in two stages: at high rates during phase reconstruction of materials up to 100 h of homogenization and much more slowly during the long-term annealing of the phase-stable structure for up to 1000 h. The observed decrease in the content of oxides in the volume of the sinters from 100–1000 h of annealing may be apparent and result from the limited resolution of the proposed image analysis method.

However, the problem of titanium oxide stability is more complex, and the rutile phase should be treated as something other than a stable, high-temperature allotropic form of the oxide.

**Figure 5:**
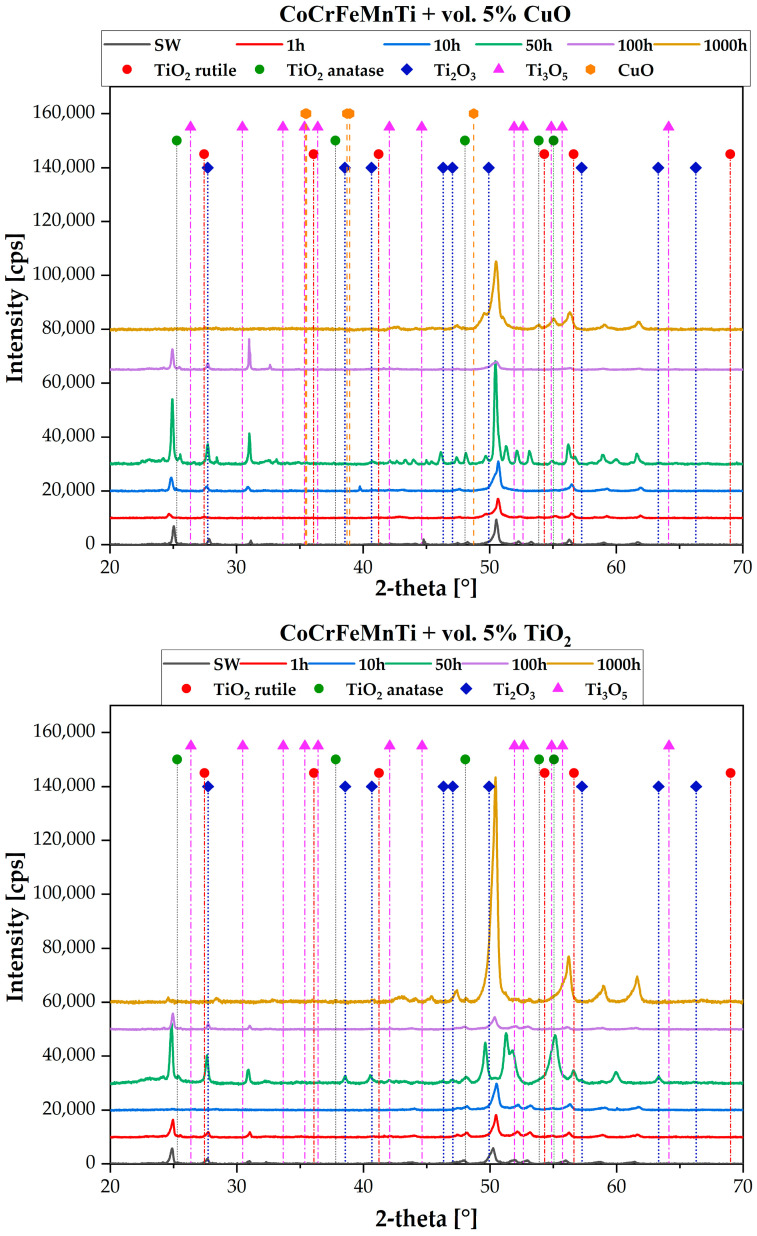
X-ray diffraction phase analysis of samples of the CoCrFeMnTi base composition with CuO and TiO_2_ additives in the as-sintered state subjected to homogenization annealing for 1, 10, 50, 100, and 1000 h, along with the positions of the planes for the following compounds: TiO_2_ in the form of anatase and rutile, Ti_2_O_3_, Ti_3_O_5_, and CuO.

To determine which specific oxides are present in the structure of the sinters, X-ray phase analysis was performed. During this analysis, the designed alloys were characterized by a two-phase matrix composed of a solid solution with a BCC lattice and a C14 intermetallic phase with an increased content of titanium. However, considering that the oxides were deliberately introduced into the alloys, an attempt was made to identify them. Unfortunately, the cards corresponding to TiO_2_-rutile: 00-021-1276, anatase: 00-021-1272, CuO: 00-041-0254, or the equally often analyzed titanium oxides, Ti_2_O_3_: 00-010-0063, and Ti_3_O_5_: 00-010-0063, did not match any of the alloys, neither for the diffractograms corresponding to the states of the alloys heated for 1 nor for those heated at 1000 h (Figure 5).

**Figure 6:**
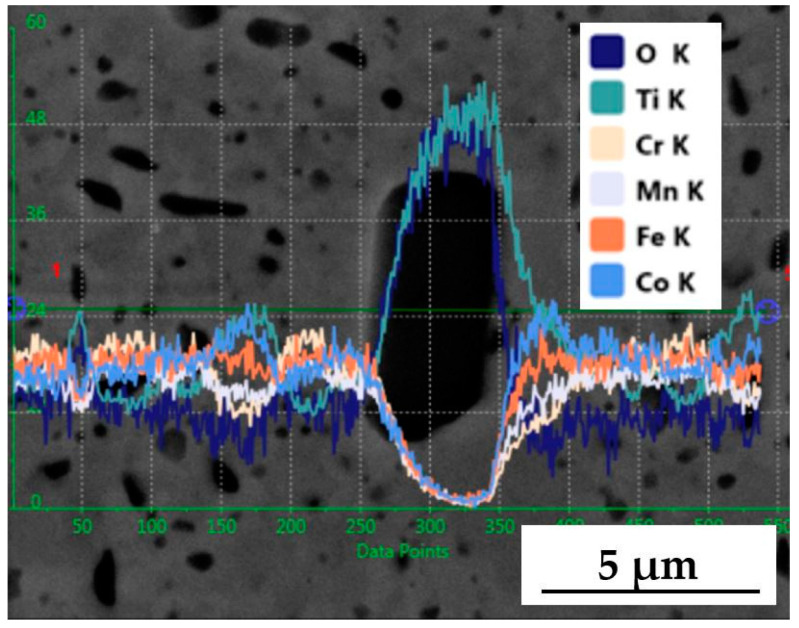
Linear EDS analysis of the structure of the TiCoCrFeMn alloy with the addition of 5 vol.% TiO_2_ in the area of titanium oxide precipitation.

Therefore, the next step was EDS analysis. As shown in Figure 6, the content of titanium and oxygen increases in areas of the characteristic oxide precipitations. This confirms the presence of titanium oxide, but it is impossible to determine the exact allotropic form of the compound or the oxidation state of the essential element, Ti.

**Figure 7:**
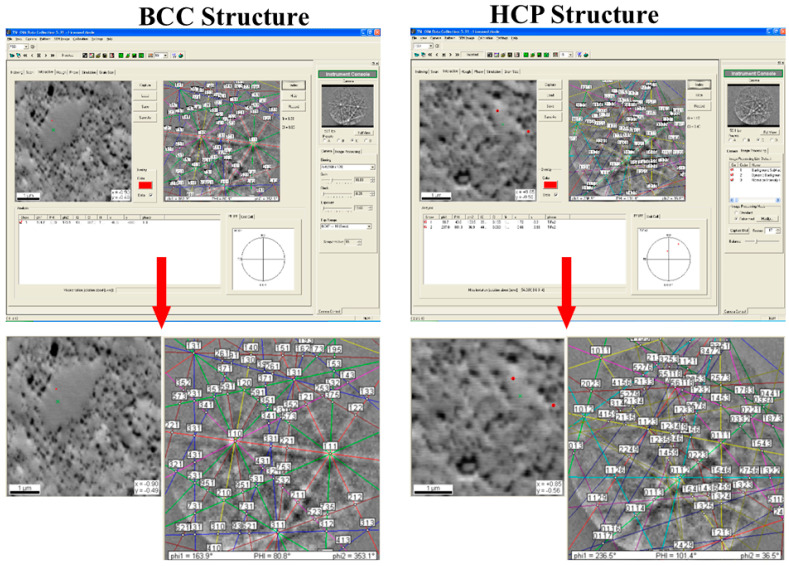
Identification of the phases forming the matrix of the tested materials, the BCC and HCP lattices, via EBSD.

Despite confirming the presence of an increased content of oxygen and titanium in the area of the precipitate subjected to analysis, the exact type of phase consisting of both elements cannot be determined. However, owing to the unavailability of a transmission electron microscope, a scanning electron microscope with a built-in EBSD camera was used for this purpose. The area for EBSD analysis, the matrix phase, was identified using the chosen method (Figure 7), and an attempt was made to estimate the type of phase representing the oxide precipitates. XRD studies show that high-entropy alloys are two phases composed of a solid solution with a simple BCC crystallographic lattice and a hexagonal C14 intermetallic phase. The solid solution is stabilized by elements with a native cubic spatially centered lattice, i.e., manganese and chromium. In contrast, elements with an HCP lattice, i.e., titanium and cobalt, stabilize the intermetallic phase.

**Figure 8:**
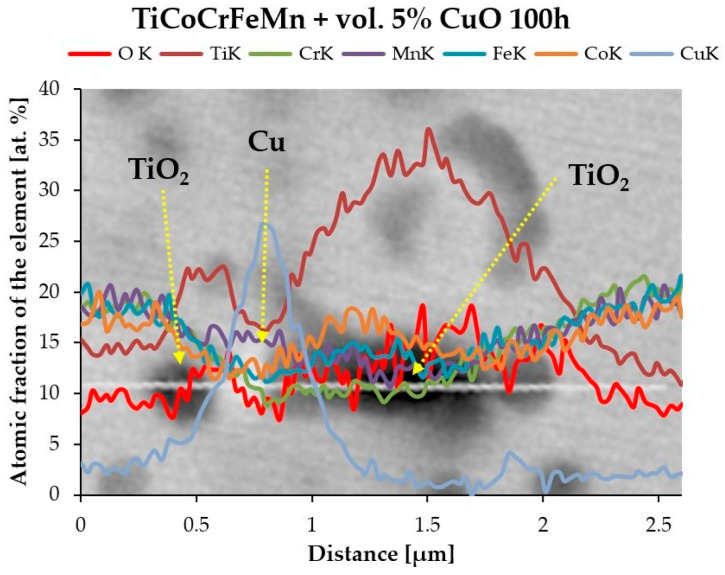
Linear EDS microanalysis of the chemical composition of the Ti oxide TiO_2_ formed near the copper precipitate in the TiCoCr-FeMn alloy with the addition of CuO heated for 10 h.

Near the oxide, there is a matrix with a compact hexagonal lattice. This probably results from the highest concentration of titanium atoms near the precipitate, which, together with the atoms of the other elements, create a phase with this crystallographic structure. Notably, however, in the case of the alloy with the addition of CuO, this oxide was reduced to Cu during alloy production, and TiO_2_ was formed. The evidence for this phenomenon is the linear microanalysis of the chemical composition performed in the oxide areas, where pure copper precipitates were found near the oxides (Figure 8).

However, it has not yet been possible to answer the following question: What allotropic form do the oxides take in the structure of the obtained sinters? The most common form of titanium oxide is the stable rutile phase. However, according to [18,19], rutile is practically the only oxidation product up to a temperature of 650 °C, and with increasing temperature, other titanium oxides are formed, including the dominant Ti_2_O_3_ with a corundum structure. Above 800 °C, TiO_2,_ Ti_2_O_3_, TiO, and Magnelli oxides are also observed. The authors of [18] conducted oxidation of a high-temperature Ti_6_Al_4_V alloy and confirmed that, above 700 °C, the corrosion products were TiO, Ti_2_O_3_, and Ti_3_O_5_ oxides. By comprehensively analyzing the above information in combination with the literature [20,21], it can be assumed that anatase and rutile phases transform into Magnelli phases and that Ti_3_O_5_, which is superconductive at temperatures above 3 K, transforms into titanium corundum Ti_2_O_3_.

**Figure 9:**
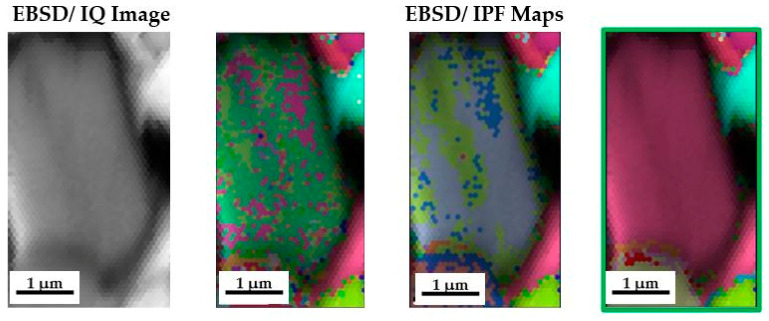
An attempt to fit anatase, rutile, and Ti_2_O_3_ to the region of the ceramic precipitate is shown in Figure 6, together with an analysis of the CI fit factor.

The above facts necessitated a more precise identification of the oxide shown in Figure 6 while providing a basis for discussing the type of oxides in the tested materials. Using the EBSD method, cards corresponding to TiO_2_ in anatase, rutile, and Ti_2_O_3_ phases were obtained, and the corresponding degree of matching is shown in Figure 9.

In addition to the visual assessment of the phase-matching accuracy, the CI (confidence index) was taken from the help manual of the Team Edax program in which the phase identification maps were made. This parameter is calculated by automatically indexing diffraction images. In practice, the CI values range between 0 and 1, and two analysis intervals are considered:

- CI < 0.1 means a low degree of matching of lines to cards and a small fraction of correctly indicated points;
- CI = 0.1 is the accepted threshold of acceptability of phase matching (recognition).

Assessing the declared oxides on this basis, it was revealed that the worst match was characterized by anatase (CI < 0.1). The rutile phase was matched to an acceptable degree, reaching a value of CI = 0.122. However, among the three analyzed oxides, the best match was characterized by Ti_2_O_3_ oxide, for which the highest degree of match was achieved at the level of CI = 0.343, confirming that this result is reliable and consistent with the literature data.

**Figure 10:**
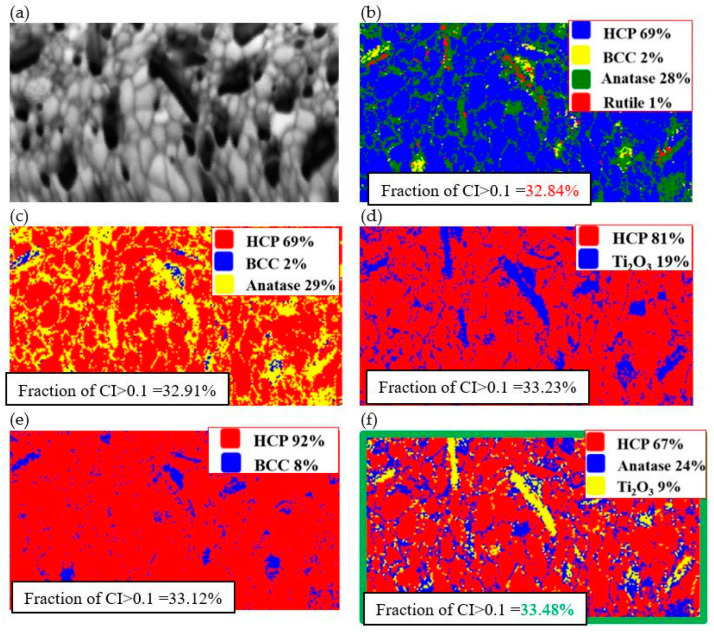
Comparison of EBSD diffraction tests aimed at identifying oxide phases for the TiCoCrFeMn + 5 vol.% TiO_2_ alloy after 1000 h of annealing in the area of Laves phase occurrence. Changes in the CI coefficient values of the analyzed structure: (**a**) when fitting the crystal lattice of BCC, HCP, anatase, and rutile; (**b**) BCC, HCP, and anatase; (**c**) HCP and Ti_2_O_3_; (**d**) HCP and BCC; and (**e**) HCP, anatase, and Ti_2_O_3_ (**f**).

Owing to the ambiguous match of oxide phases in Figure 9, resulting from the large size of the oxides and thus their easier identification, EBSD microdiffraction maps were constructed for the selected area of the alloy with the addition of TiO_2_ after 1000 h of annealing (Figure 10).

The analysis carried out in the area of the Laves phase, illustrated in Figure 10, revealed that the best match is characterized by the configuration shown in Figure 10f, which consists of 24% anatase with 9% Ti_2_O_3_ in a hexagonal matrix of the intermetallic phase. Attention should be given to the rod-shaped morphology of the Ti_2_O_3_ oxides, which was also observed via microscopy, and the high content of anatase with the complete absence of the expected rutile. However, the formation of the identified TiO_2_, Ti_3_O_5_, and Ti_2_O_3_ oxides is also thermodynamically justified.

These forms of oxides are characterized by the following values of the change in the standard Gibbs energy as a function of temperature [34]:

$${\mathrm{TiO}}_{2}\text{: }Ti+O_{2}={TiO}_{2},\;\Delta G{}^{\circ}=-\text{217,703}+41.4\text{ T cal/mol}$$

$${\mathrm{Ti}}_{3}O_{5}\text{: }\frac{6}{5}Ti+O_{2}=\frac{2}{5}{Ti}_{3}O_{5},\;\Delta G{}^{\circ}=-\text{232,950}+40.9\text{ T cal/mol}$$

$${\mathrm{Ti}}_{2}O_{3}\text{: }\frac{4}{3}Ti+O_{2}=\frac{2}{3}{Ti}_{3}O_{3},\;\Delta G{}^{\circ}=-\text{239,166}+41.2\text{ T cal/mol}$$

**Figure 11:**
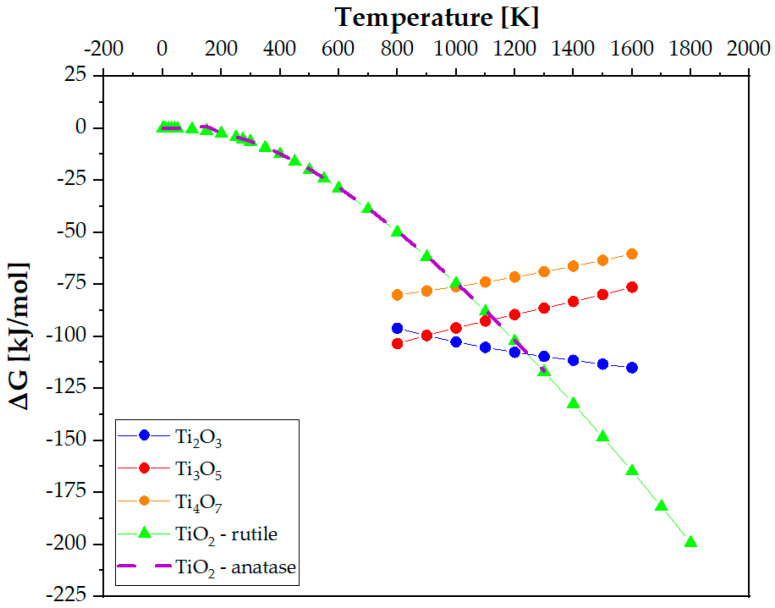
Gibbs free energy values for the oxides: TiO_2_ (in the form of rutile and anatase), Ti_2_O_3_, Ti_3_O_5_ and Ti_4_O_7_.

After considering the temperature factor, we notice that comparing the Gibbs energy values as a function of temperature favors the formation of the lowest energy oxide, Ti_2_O_3_, first and then only Ti_3_O_5_ and TiO_2_ (Figure 11). The difference in the free energy of anatase and rutile phases is very small and is only 2.3 kJ/mol or less at all temperatures. However, anatase is more likely to form in the presence of Ti [30,35], which would explain the lack of rutile in the materials studied.

**Figure 12:**
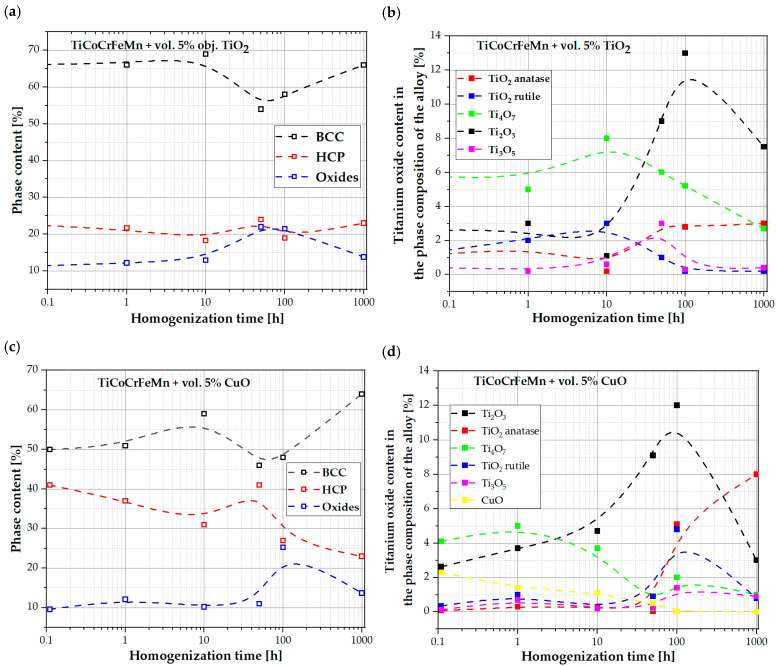
Percentage changes in the content of the main phases (BCC, HCP and Oxides) in alloy with TiO_2_ (**a**) and with CuO (**c**) and titanium oxide content changes in the phase composition of the alloy with TiO_2_ (**b**) and with CuO (**d**) of the obtained sinters with increasing annealing time.

The conducted analyses allowed us to determine the content of individual allotropic varieties of titanium oxides in the structure of the annealed sinters (Figure 12). After 1000 h of annealing at 1273 K, the largest content of oxides in the structure of the sinters consisted of Ti_2_O_3_ and anatase oxides. This effect was more visible as more energy was supplied to the system. This was the case with the addition of CuO, which was transformed into TiO with the participation of an exothermic reaction and the release of additional energy.

## 4. Conclusions

Analysis of the changes in the oxide content during long-term annealing at 1323 K is a complex issue. Understanding these changes requires an assessment of the possibility of oxide reduction in the presence of intermetallic phases containing titanium and cobalt, as well as investigations into the thermodynamic mechanisms of titanium oxide reconstruction in the presence of multicomponent high-entropy phases.

1. In the produced TiCoCrFeMn alloys with additives of at. 5% CuO and at. 5% TiO_2_, a significant share of oxide precipitates was observed, which coagulated and grew as a function of homogenization time. The analysis showed an increase in the share of oxides up to 100 h of homogenization, and a decrease in their share from 100 to 1000 h.
2. TiO_2_ oxides formed during the process as a result of the reconstruction of the CuO oxide introduced in situ are more coherent with the matrix components and grow more slowly than TiO_2_ introduced ex situ.
3. Attempts were made to identify the oxides using X-ray phase analysis, which, unfortunately, did not clearly confirm the presence of TiO_2_ in the form of rutile/anatase, CuO, or even common oxides with negative Gibbs energy values, such as Ti_2_O_3_ or Ti_3_O_5_. However, EDS analysis was used at the stage of microscopic observations, which only allowed for the identification of titanium oxides without the possibility of determining their exact allotropic form. In the alloy with the addition of 5% CuO, the presence of pure Cu was determined near titanium oxides, confirming the reduction of CuO to Cu and TiO_2_.
4. Due to the above problem, it was decided to use the EBSD methodology to try to identify the exact allotropic forms of oxides. Based on samples heated for 1000 h (containing oxides of the largest sizes), it was determined that the best match corresponded to a configuration of 24% anatase and 9% Ti_2_O_3_ in the HCP matrix. The presence of rod-shaped Ti_2_O_3_ oxides and a high proportion of anatase was observed in the absence of the expected rutile. However, the literature analysis explains the lack of rutile and the formation of anatase in the presence of Ti.
5. Additional analysis confirmed the highest share of anatase and Ti_2_O_3_, especially in the alloy with the addition of CuO, in which this oxide was transformed into titanium oxide through an exothermic reaction, releasing additional energy.

The acquired knowledge will fill the gap in the scope of the selected new group of materials, which are further strengthened by ODS oxides, offering perspectives for applications in nuclear energy and a substitute for the known ODS martensitic steels.

## Data Availability

The raw data supporting the conclusions of this article will be made available by the authors on request.
